# Effectiveness of Multicomponent Intervention on Quality of Life of Family Caregivers of Cancer Patients

**DOI:** 10.31557/APJCP.2021.22.9.2789

**Published:** 2021-09

**Authors:** Malathi G Nayak, Anice George

**Affiliations:** 1 *Department of Community Health Nursing, Manipal College of Nursing, Manipal Academy of Higher Education, Manipal. Karnataka, India. *; 2 *Department of Paediatric Nursing, Manipal College of Nursing, Manipal Academy of Higher Education, Manipal. Karnataka, India. *

**Keywords:** Caregivers, quality of life, awareness programme, education, pranayama

## Abstract

**Objective:**

The study aimed to determine the effectiveness of the multicomponent intervention on Quality of life (QOL) of family caregivers of cancer patients.

**Methodology:**

A Quasi-experimental study with pre and post-test measures was conducted among 200 caregivers of cancer patients selected by convenient sampling technique. The experimental group received the intervention, and no intervention was given for the control group during the study period. The data were collected from family members looking after the cancer patients diagnosed with breast/head & neck cancers and who were in the third and fourth stages of cancer. After the pre-test, provided multicomponent intervention (Pranayama, yogic relaxation, counselling and Education) and a post-test was conducted at the first month & third month. The obtained data were analysed and inferred using descriptive and inferential statistics to compare the outcomes among the groups.

**Result:**

Most of the caregivers belong to the age group of 31 to 40 years in the intervention group (31%) and in the control group (35%), 31% of them had their education up to a primary level in both the groups and most of the caregivers were spouses (49%). Regarding the QOL of family members, improvement was noted from baseline 66.66 to 126.82 (3 months) among the intervention group, and in the control group, mean QOL enhanced from 59.77 to 81.97. Repeated measure ANOVA was computed to analyse difference within and between the groups and it showed that the intervention program was efficacious in enhancing the QOL of family caregivers of cancer patients (F (1, 191) =639.02, p=.001).

**Conclusion:**

Nurses play a vibrant role in improving the Quality of life of family members of cancer patients since they are mostly involved in providing care. Health care system must ensure that the family members who provide care to the cancer patients receive appropriate teaching programmes on reducing their burden.

## Introduction

Family Caregivers (FCG) provide care to their beloved ones with cancer throughout his/her life, and they will have many concerns while caring for the patients with disease. Hence, the caregivers also experience burden, which affects their Quality of life. However, while looking after the cancer patients, family caregivers often experience psychophysical and social distress, limitation of roles and activities, and strain in marital relations (if the caregiver is the spouse). In progressive disease, caregivers perform a vibrant task in providing support with day-to-day activities, medications, bringing the patients for a check-up and advocating the patients’ concern to the experts. All phases of the health of caregivers get affected in caring for cancer patients (Given, 2005). The caregiving burden was mainly due to anxiety, needing time and energy to access the health services, community resources, and obtaining health information (Farrell, 2011). Family caregivers experience a significant amount of distress while providing care with limited available resources (Farrell, 2013). When the care provider’s requirements are considered, and training is delivered, the outcome of care offered is reported to be improved. Most cancer patients have cared at home without formal home care services (Emanuel et al., 2000). Cancer disease itself brings changes among cancer patients in all dimensions. These changes also affect caregivers’ quality of life while trying to meet all the cancer patient’s needs. Family care providers of cancer patients listed various psychosomatic sufferings; that affects their overall Quality of life (Stenberg, 2010; Romito, 2013). Due to their caregiving responsibilities, most caregivers neglect their own needs (Beesley et al., 2011; Molassiotis et al., 2011). During hospitalisation, family caregivers offer care to the cancer patients throughout their stay; hence they are ignored about their own wellbeing (Rhaet al., 2014). However, to provide adequate attention to the cancer patients, family members require an awareness package on managing symptoms and medication, coping abilities and financial support (Given et al., 2001; Yun et al., 2005). Psychoeducational interventions and therapeutic counselling help to improve QOL, and strategies are to be developed for training the caregivers to decrease their burden and improve the QOL in meeting the demands of the cancer survivor (Kim andYi, 2015; Guerriere et al., 2015; Turkoglu and Kilic, 2012). More integrated, comprehensive approaches are to be offered to maintain the coping abilities of caregivers and cancer patients (Aydogan, et al., 2016; Mosher et al., 2016; Northouse, 2005). An educational support programme is needed to enhance caregiving skills and reduce the caregiver suffering, which should be addressed in culturally appropriate/holistic cancer care (Vrettos et al., 2012; Song et al., 2011; Manir and Ghosh, 2019). Few of the other studies suggested that to reduce the caregivers’ burden and to improve the quality of life, there is a need for interventional studies to provide effective care to their cancer patients (Heidai et al., 2012; Grant et al., 2013; Rha et al., 2015). Various interventional studies have been implemented nationally and internationally to improve the quality of life of cancer patients. It is observed that interventional research studies done among family caregivers to improve their quality of life are very minimal in India. Therefore, the researchers in this study explored the possibilities to implement awareness programmes to enhance family caregivers’ quality of life in a tertiary level hospital. 

## Materials and Methods

**Figure 1:**
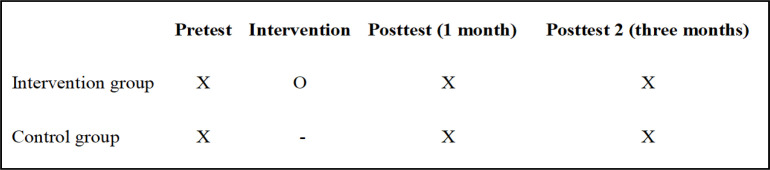
Schematic Representation of Study Design

A Quasi-experimental study with pre and post-test measures was conducted among 200 (100 experimental and 100 control group) FCGs of cancer patients selected by convenient sampling technique. The objective of the study was to assess the impact of multicomponent interventional program on the QOL of the FCG of cancer patients. To avoid sample contamination and getting the sample, the data were collected (from November 2016 to February 2019) from two tertiary care hospitals from Manipal and Mangalore after obtaining administrative permission. Both tertiary care hospitals belong to one university administration with similar infrastructure. The researcher explained the nature of the study procedure to the eligible participants and obtained informed consent. The researcher assured the privacy of the data collected and obtained the Institutional Ethical clearance from the Kasturba Medical College and Kasturba Hospital, Manipal. The inclusion criteria for caregivers were closely connected to cancer patients, most involved in the care of breast and head and neck cancer patients (at least 2-3 hour per day), aged above 20 years, know the local language and are willing to participate. The researcher identified the caregivers of patients with breast and head & neck cancer who were in the third or fourth stage of cancer and on treatment. The type and stage of cancer and their residential details were noted from the hospital registers. Before taking the consent from the caregivers, they were informed about the awareness program as an intervention and follow up requirements at one-and three-months interval. To build a rapport with the patient and caregivers, the researcher visited them twice a day before collecting the data. The intervention group received the multicomponent intervention, and the control group had regular and routine information as per the requirement Follow up was done for both the groups at one month and three months. The interview technique was used to collect the data using the (QOLLTI-F) questionnaire and demographic characteristics from the family members. The schematic representation of study design is depicted below: (Figure 1).

*Multicomponent Intervention*

The intervention composed of three sessions of 70 minutes’ duration. After obtaining the informed consent from the caregivers of cancer patients, at the opening, a pre-test was conducted, it was followed with intervention (Pranayama and yogic relaxation, counselling and education), and a post-test was carried out at first and third-month interims. The counsellor and yoga therapists were appointed under this project to counsel caregivers with low quality of life score (less than 50% score from the total score). Pranayama and relaxation technique were provided in a separate room of hospital for relaxation of body and mind.

The researcher provided an awareness program about the care of cancer patients on symptom management and informed the caregivers about the various health schemes available in India to overcome the financial burden. The intervention package was delivered on an individual basis, depending on the need of the caregivers. The intervention of pranayama, yogic relaxation, counselling, and education was given three times in the first week week. After that, the researcher made observations/follow-up on the 2^nd^, 3^rd^, and fourth week of intervention. The post-test was carried out at first and third-month interims (Table1). In-between, the researcher made frequent telephone contact with the caregivers of cancer patients to remind them to practice pranayama and relaxation regularly. At the end of the session the information booklet was provided to all the enrolled participants. 

The obtained data were coded, analyzed and interpreted using descriptive and inferential statistics to compare the outcomes among the groups based on the study’s objective. (SPSS package version 16: IBM SPSS Inc., Chicago).

*Description of the data collection tools*

The data collection questionnaire had two sections: demographic characteristics and QOL questionnaire (QOLLTI-F).

*Tool 1: Demographic characteristics*

This tool was established to acquire the background data of the FCGs. The tool comprised items on age, gender, religion, marital status, educational status, employment status, relationship to caregivers. A total of seven items were retained after content validity (CVI =. 97).

*Tool 2: Quality of Life (QOLLTI-F): for family caregivers*

The investigator used a standardized instrument from McGill University to assess the QOL of the caregivers of cancer patients after obtaining permission from the tool developer to use the tool. The QOL of the caregivers was assessed by using QOLLTI-F (Quality of Life in Life-Threatening Illness - Family Carer Version), which was developed at the Department of Palliative Care McGill University (Cohen et al., 2006). The test-retest reliability for the QOLLTI-F total score was 0.78 for the seven subscale scores. The subscales were environment, patient status, caregivers’ own status, caregivers’ outlook, Quality of care, relationship, and financial worries. Under each subscale, there were sub-items. They were environment (2), patient state (1), caregivers’ own state (5), caregivers’ outlook (3), Quality of care (2), relationship (2), and financial worries (1). The total score of QOLLI-F was the mean of the seven subscale scores and the score ranged from 0 - 160. Higher the score better the Quality of life. Since it was a standardized tool, it was translated into the local language, and reliability was obtained. The reliability coefficient obtained was r = 0.80.

## Results

At baseline 200 family caregivers were enrolled to the study and baseline (pretest) data were collected. Out of 200 caregivers, 193 caregivers completed follow-up assessments at one and third month and constituted the final sample for analysis (97 in the experimental group and 96 in the control group). A total of seven caregivers dropped out from the study during follow up because of the death of their patients. 

*Description of sample Characteristics of family caregivers*

Out of 200 FCGs, 31% and 35% of them were in the age group of 31 to 40 years in both intervention and control groups, respectively, and the mean age was 41yrs among the groups. The majority were females in both the groups (intervention 55% and the control group 67%), most of them had a primary level education in both the groups (31%), and most of them were spouses giving care to their dear one during treatment (Table 2).

*Description of QOL of family caregivers*

The data of the QOL of 200 FCGs from the intervention group and control group were obtained by using the QOLLTI-F tool. The higher the score better is the Quality of life.

The mean and standard deviation on sub-areas of QOL of the family caregivers showed that their own status were distressed. Their association with other people was disturbed, and their economic situation was challenging due to the patient’s disease condition (Table 3). The data depicted in Table 3 indicates that after awareness program intervention, there is a mean difference of QOL between two groups across the period. In the intervention group, the mean QOL improved from baseline 66.66 to 126.82 in the three months follow up and in the control group, QOL improved from baseline 59.77 to 81.97 in the three months follow up. Therefore, it is observed that an awareness program was apperently effective in improving the QOL of family caregivers in the intervention group compared to that of control group (Table 4).

*Effect of the multicomponent interventional program on QOL scores between and within the groups of Family Caregivers*

To determine the significance in difference within and between the groups of caregivers on QOL, a repeated-measures ANOVA followed by post hoc test using Bonferroni analysis was computed.

The data in Table 5 demonstrate that the mean QOL score was significantly different within the group (F (1.8, 191) =20664.15, p< 0.001) using Greenhouse – Geisser correction. The repeated measures ANOVA between the intervention and control groups show a statistical significance, F (1, 191) =639.02, p=.001). This shows that the multicomponent interventional program was effective in improving the QOL among family caregivers in the intervention group compared to that of control group. Further, to find out the significance difference in the means, a pairwise comparison was made using Bonferroni comparison for multiple comparisons. The pairwise comparison is shown in Table 6. 

**Figure 2:**
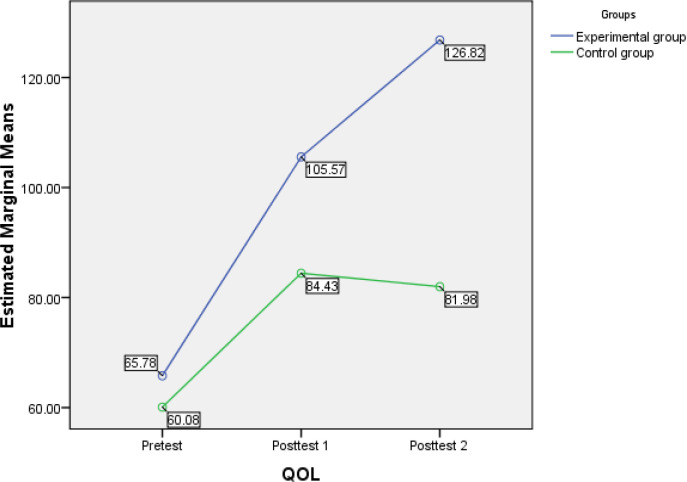
Mean Plots Showing the Difference in the Estimated Marginal Means of Post-test of QOL at one and 3 Months of Intervention

The post hoc test using Bonferroni analysis revealed that the awareness program intervention resulted in statistically significant improvement in QOL between pre-test and post-test 1 (p<.001) and pre-test and post-test 2 (p <.001) of caregivers. Thus, it is concluded that the multicomponent interventional programme and the education of caregivers for three months can significantly improve their QOL and their knowledge in giving care to cancer patients. The significance difference in the mean is plotted in Figure 2. This result implies that differences in the score across the time could be attributed to the effect of the multicomponent intervention has the positive effect on the caregivers QOL. 

**Table 1:** Frequency of Multicomponent Intervention

Type of Interventions	Duration	Intervention 3days /week (1^st^ week)			Observation and follow up				Follow up
For caregivers of cancer patients:		Day1	Day 2	Day 3	2^nd^ Week	3^rd^ Week	4^th^ Week	One month	Third month
Yoga (Pranayama and relaxation)	20 mts per day	+	+	+	+	+	+		
Counseling session	20 mts individually/ session	+	+	+	+	+	+		
Awareness Progrmme	30 minutes	+	+	+	+	+	+	Post test 1	Post test 2

**Table 2:** Frequency and Percentage Distribution of Caregivers Based on Demographic Characteristics

Sample characteristics	Intervention group (N=100)		Control group (N=100)	
	F	%	f	%
Age in years				
20 - 30	25	25	22	22
31 - 40	31	31	35	35
41 - 50	22	22	28	28
Above 50	22	22	15	15
Gender				
Male	45	45	33	33
Female	55	55	67	67
Religion				
Hindu	93	93	83	83
Christian	3	3	5	5
Muslim	4	4	12	12
Educational status				
Illiterate	19	19	25	25
Primary	31	31	31	31
High School	16	16	25	25
Pre University	8	8	8	8
Graduate	22	22	9	9
Post Graduate	4	4	2	2
Marital status				
Married	87	87	87	87
Unmarried	13	13	12	12
Widow	-	-	1	1
Relationship				
Spouses	49	49	49	49
Child	29	29	26	26
Parents	6	6	6	6
Siblings	11	11	13	13
Others	5	5	6	6
Employment status				
Employed	26	26	16	16
Unemployed	32	32	28	28
Retired	5	5	3	3
Housewife	37	37	53	53

**Table 3:** Mean and Standard Deviation of Pre-test QOL Scores of Intervention and Control Group of Family Caregivers on Seven Sub-areas (N=200)

		Intervention group	Control group	Intervention group	Control group
Subareas	Possible Score	Mini (max)	Mini (Max)	Mean (SD)	Mean (SD)
Environment	20	2 (18)	2(18)	9.26 (3.02)	8.51 (3.70)
Patient state	10	0 (9)	1 (7)	2.78 (1.80)	1.91 (1.36)
Carer’s own state	50	5 (43)	5 (31)	14.59 (7.09)	12.54 (5.28)
Carer’s outlook	30	4 (30)	3 (23)	15.48 (5.43)	14.11 (5.51)
Quality of care	20	7 (20)	12 (19)	17.38 (1.51)	17.39 (1.17)
Relationships	20	2 (18)	1 (16)	5.53 (3.90)	4.16 (2.60)
Financial worries	10	0 (8)	0 (5)	1.65 (1.50)	1.15 (.74)
Overall QOL	160	36 (140)	32 (98)	66.66 (17.30)	59.77 (12.93)

Mini, Minimum score; Max, Maximum score; SD, Standard Deviation

**Table 4:** Mean and Standard Deviation of Pre and Post-test QOL of Intervention and Control Group of Family Caregivers (N=200)

	Intervention group		Control group	
QOL	Mean	SD	Mean	SD
QOL Pretest	66.66 (N=100)	17.30	59.77 (N=100)	12.93
QOL Posttest 1 ( At one month)	105.60 (N=99)	8.7	84.41(N=98)	6.58
QOL Posttest 2 (At three months)	126.82(N=97)	11.41	81.97 (N=96)	9.20

SD, Standard Deviation

**Table 5:** Repeated Measures ANOVA on QOL Scores within and between Groups of Family Caregivers (N=193)

QOL	Mean square	F Value	Df	P	η^2^ *p*
Within the group (N=97)	20664.15	148.5	1.8, 191	0.001	0.437
Between the group (N=193)	82647.39	639.02	1, 191	0.001	0.770

Note, df , degree of freedom; η^2^ *p, *partial eta (effect size).

**Table 6:** Pairwise Comparison of QOL Family Caregivers (N=200)

Mean Measurement		Mean Difference	Standard Error	Significance*	95% confidence interval difference	
					Lower bound	Upper bound
Pre-test	Post-test 1	-32.06	1.2	0.001	-34.96	-29.16
Post-test 1	Post-test 2	-9.4	0.949	0.001	-11.69	-7.11
Pre test	Post-test 2	-41.46	1.25	0.001	-44.5	-38.42

Based on estimated marginal means; *The mean difference is significant at the 0.05level an Adjustment for multiple comparisons: Bonferroni.

## Discussion

The present study evaluated the effectiveness of multicomponent intervention among the FCGs of cancer patients. This study shows that the mean age of the caregivers was 41 years, and the majority of the caregivers were female (55% in experimental and 67% in control group) and were spouses (49%). These results are supported by Guerriere et al., (2015) found in their study the mean age was 59 years, and 70% were females. Another study was done among 200 cancer fighters and their care providers in New Delhi. The outcomes showed that most caregivers were females (55%), the mean age was 40 years, unemployed (51.5%), and 27% were illiterates, 57.5% were spouses (57.5%), and around 43% of the family caregivers lost their job due to the patient’s sickness (Lukhamana et al., 2015). 

With regard to quality of life of family caregivers, the present study observed that the mean score of QOL at baseline was 66.66 in the intervention group and 59.77 in the control group; Comparable outcomes were detected in a study done by Kim et al., (2015) in Korea among 191 family caregivers by using the Korean version of the Caregiver QOL Index-Cancer questionnaire, where the mean QOL score was 74.62. A negative correlation was found among the unmet needs of the family caregivers and their QOL (Kim and Yi, 2015; Guerriere et al., 2015; Turkoglu and Kilic, 2012). A similar QOLLTI-F tool was used to assess the QOL of family caregivers in Kolkata by Pal (2019) and Nayak et al., (2014) observed that more than fifty percent of the caregivers had poor Quality of life. 

The majority of the Indian population belongs to below the poverty level. There is a necessity to prioritize the decrease of economic liability by giving awareness of the availability of schemes provided by the government. The present study shows that the family caregivers’ faced financial problems due to disease condition of their loved one and their relationship with others affected by caring; similar findings are reported in a study conducted in Istanbul, which reported that due to the caregiving concerns their routine activities were affected (53.3%) (Yakar and Pinar, 2013). They also faced difficulties in their entire work-life (30%) and family relations (15%); no financial support received from other family members was the causative factors for the low Quality of life of family caregivers life (Yakar and Pinar, 2013; Yun et al., 2005; Nayak et al., 2018; Kizza and Muliira, 2019). 

The present study showed that the awareness program was beneficial in terms of improved QOL of caregivers from baseline data to follow up at three months when compared between the groups across the time period. Family caregivers also expressed that their caregiving experience improved, which facilitated them to become better care providers to the care recipient as well as enhanced their Quality of life. This finding concurs with that of Cagle et al., (2015) who used Effective Management of Pain: Overcoming Worries to Enable Relief (EMPOWER) programme among 126 family caregivers of four hospice centres in USA to empower the family caregivers. The patients and their family caregivers had expanded their knowledge on symptom management and reduced their concerns after two weeks of the intervention. Meta-analysis was done on types of interventions used among family caregivers to improve their Quality of life; pooled data showed that psychoeducational, skills training and therapeutic counselling had a significant reduction of care burden and improved their Quality of life and also authors suggested there is a need of interventional studies among caregivers on a larger sample (Northouse, 2010). Gabriela and Mayers (2019) has observed in their study a reduction in caregivers burden after psychosocial intervention in Nigeria (T1:p=0.000, T2:p=0.018) and improved QOL when compared between the groups (p=0.000, p=0.020 respectively). Few other studies utilized various interventional programmes like Creativity, Optimism, Planning, and Expert information (COPE), Caring for the Caregiver Programme (CCP), Family involvement, Optimistic attitude, Coping effectiveness, Uncertainty reduction, and Symptom management (FOCUS) and Comprehensive Health Enhancement Support System (CHESS) to improve the quality of life of caregivers and reported as there is a positive significant effect on burden reduction and improved the QOL of the caregivers (Belgacem et al., 2013; Bahrami and Farzi, 2014; Leow et al., 2015; Northouse, 2013; DuBenske, 2014). However this study recommended that equal importance to be given to the family caregivers as well cancer patients to improve their quality of life.

*Strength of the study*

In this study we found that non pharmacological interventions were beneficial in reducing the stress and bringing relaxation among the FCGs of cancer patients. Family care givers were empowered with the information regarding care of cancer patients and the facilities/ financial support available for the same.

*Weakness of the study*

As the study design used a non-randomised design it is acknowledged that the strength of the study is limited. Study included only caregivers of breast and head & Neck cancers, study cannot be generalized to caregivers of all types of cancers.

In conclusion, in the present study, the multicomponent interventional programme (pranayama, and yogic relaxation, counselling and education) was provided as an intervention on an individual basis in view of improving the Quality of life of caregivers, and it was found effective. Therefore, it is imperative to use non-pharmacological methods to reduce the distress and burden among caregivers. QOL is an individual’s subjective perception of wellbeing and coping ability. Therefore, there is a need for active support on the issues of caregiving burden. It is essential to have a comprehensive cancer care program for patients and their caregivers at the time of diagnosis to sustain their health condition and improve their Quality of life. 

## Author Contribution Statement

MGN developed protocol in consultation with AG. MGN and AG equally contributed to the study design. MGN responsible for the data collection, data management and statistical analysis. AG made critical revision to the paper. Both authors have contributed to refinement of the study protocol and approved final manuscript.
